# Supplementary material for: Are carbon emissions trading and green financial instruments synergistic? -Comprehensive quantitative research based on content analysis
Source: PLoS One. 2024 Mar 7;19(3):e0298601. doi: 10.1371/journal.pone.0298601 (PMC10919601; doi:10.1371/journal.pone.0298601)
Supplement: S1 Data — (ZIP) [file pone.0298601.s001.zip › Data/地级市Z_F工作报告.zip!/░▓╗╒/░÷▓║╩╨/└┤╫╘╣1⁄2╓┌║┼í╛┬φ┐╦╩2╛▌═°í┐_2008.docx]

各位代表：
现在，我代表市人民政府向大会报告工作，请予审议，并请市政协委员和其他列席人员提出意见。
    一、2007年和过去五年工作回顾
刚刚过去的2007年是不平凡的一年，也是我市经济社会继续保持又好又快发展的一年。在中共蚌埠市委坚强领导下，全市上下积极应对宏观调控新形势，努力克服淮河流域1954年以来特大洪水造成的不良影响和其它困难，求真务实，开拓进取，较好地完成了市十三届人大五次会议提出的目标任务。
——国民经济较快发展，主要目标全部实现。预计全年实现地区生产总值410亿元，比上年增长13%左右，其中一、二、三次产业增加值分别增长5%、16%和12.5%。实现财政收入56.2亿元，增长23.7%，当年净增额突破10亿元；地方财政收入22.3亿元，增长32.4%。固定资产投资完成185亿元，增长31.5%。进出口总额3.07亿美元，增长11.7%。实际利用外资2亿美元，增长39.8%，引进内资115亿元，增长48%。社会消费品零售总额156亿元，增长16.5%。城镇居民人均可支配收入11200元、农民人均纯收入3600元，分别增长10.1%和13.5%。居民消费价格上涨5%左右。
——工业保持快速增长，运行质量有所提升。深入推进工业强市战略，积极实施“8765”工业振兴工程，做大工业经济总量。壮大骨干企业取得进展，规模以上工业完成总产值309亿元，增长19.8%；实现增加值101亿元，增长18.4%。工业运行质量较好，工业经济效益综合指数达170%。加快工业企业改组改制，盘活存量资产。引导企业退市进园，支持各类园区错位发展、集约发展，园区实现产值125亿元。大力推进自主创新，高新技术产业比重提高1.1个百分点，金威公司BB牌内燃机滤清器、天润公司聚丙烯酰胺荣获中国名牌产品称号。部分工业企业实施“二次创业”取得新的突破。
——新农村建设继续推进，县域经济发展加快。奋力夺取抗洪救灾全面胜利，农业生产保持稳定，粮食总产和单产再创历史最好水平。调整优化农业和农村经济结构，加快农业产业化进程，新增省名牌农产品9个。65万亩绿色食品原料标准化生产基地通过国家认定。加大劳务输出力度，转移农村劳动力57万人。105个新农村建设试点村规划全部完成，72个新农村建设项目顺利实施。积极改善农村生产生活条件，新建和改造农村公路860公里，搬迁改造“空心村”8个，建设农田水利工程126处，完成淮河行蓄洪区移民迁建1万户。着力提升县域经济发展水平，县域地区生产总值、财政收入和固定资产投资增幅均创历史最好水平。 
——完善商业基础设施，服务业水平继续提高。深入实施跨河发展、“双核”带动商业发展战略，打造老城区、新城区、淮上新区三大商圈。家乐福、国美电器蚌埠店正式运营，通成国贸、路桥市场一期投入使用。创新城乡流通方式，推进社区“双进”工程、“万村千乡”市场工程。巩固传统商贸业，培育汽车、通讯、旅游等新型消费热点，推动银行、证券、保险业发展。举办了涂山禹王庙会，开发和实施了11个旅游项目，实现旅游总收入17.6亿元，增长37%。房地产业发展加快。
——努力加大项目投入，固定资产投资增长较快。调整充实“十一五”项目储备库，扩展了20亿元的工业投资项目库，实施亿元以上项目15个、千万元以上工业项目150个。八一扩建、500千伏蚌埠输变电、海螺水泥粉磨站等20个项目建成投产，国电蚌埠电厂、北泰汽配工业园南区、淮北大堤除险加固、蚌埠闸船闸扩建等40个项目开工建设。城市环境综合整治世行项目、水环境综合治理日元项目等国外贷款项目通过评估。京沪高速铁路蚌埠段、合蚌客运专线、大庆路淮河大桥等一批重大项目的前期工作稳步推进。
——继续强化招商引资，对外开放成效明显。创新招商思路和方式，推行驻点招商、园区招商、以商招商，先后举办浙商合作恳谈会、中国光彩事业促进会重点联系地区年会、房地产招商会等一批重大招商活动，招商引资数量和质量实现新突破，全年共引进项目342个。大力发展外经外贸，扩大中东、非洲等新兴市场出口，出口产品结构明显优化，实现对外经济合作营业额7000万美元。
——坚持建设与管理并重，城市面貌继续改善。土地利用总体规划修编工作正在推进，龙子湖西部开发、姜桥物流工业园区等12个重点地块的专项规划基本完成。21个房屋拆迁遗留项目拆迁完毕，大板楼危房改造1600多户房屋拆迁任务2个多月完成。全面调整拆迁补偿政策，启动了新一轮棚户区和旧住宅区改造工程。市区南、北、西三大出口改造正在推进，其中解放路南段、燕山路东段改造进展顺利，省道101南段主路面改造全面完成，涂山路与黑虎山路交叉口改造已经征地拆迁。黄山大道建设方案已经通过。淮河路等“四路一桥”综合整治全面实施。龙子湖南公园已经竣工。东海大道景观带建设基本完成。大禹文化广场二期工程等园林新项目开工建设。张公山大塘综合治理、城市污水管网建设进度加快。以完善规章、健全机构、改革机制、专项治理等为重点强化城市管理，市区环境卫生、交通秩序、饮食摊点、“四小”车辆整治等取得新的进展，拆除违法建筑力度加大，市容市貌继续改观，创建省级文明城市、文明县城工作较为顺利。
——大力推进节能减排，环保工作得到加强。积极控制主要污染物排放，推进循环经济发展，二氧化硫和化学需氧量排放总量得到削减。加强节能工作，推进可持续发展，万元地区生产总值能耗下降4%。以解除“流域限批”为契机，深入开展环境保护专项整治，解决了一批长期未得到解决的环境问题。加强在线监控，对21家重点污染企业挂牌督办，关闭了部分污染严重企业。完善园区和项目环保评价。建立健全环保制度，加强环保能力建设，严肃处理了一批污染事故责任人。三座县城污水处理厂基本建成，市第二污水处理厂开工建设，第三污水处理厂即将竣工。露天堆放多年的西部垃圾场得到处理，八里沟等沟渠整治初见成效，鲍家沟地区部分农民即将喝上自来水。
——社会事业协调发展，民生工程积极推进。开展产学研对接活动，国家专利技术蚌埠交易中心挂牌，实施国家星火项目9项。落实义务教育经费保障机制，制定了促进义务教育均衡发展意见，蚌埠学院本科办学正式挂牌，民办蚌埠经济技术职业学院建成招生。花鼓灯舞蹈《鼓乡情韵》在第四届CCTV电视舞蹈大赛中获得一等奖，歌曲《好一个花鼓灯》获中宣部“五个一”工程奖，禹墟发掘等考古事业取得较大进展。市儿童医院挂牌，市紧急救援中心等卫生设施建设继续推进，新建改建72所乡村卫生院（室）、27个社区卫生中心（站）。成功举办市第十二届运动会，市体育场维修改造加快实施，市龙湖体育馆开工建设。新一代天气雷达、淮河流域气象中心和气象科普公园基本竣工。广播电视发射塔开工建设。妇女、儿童、老年人、残疾人权益得到保障。人事、统计、档案、外事、侨务、地震、国防动员、人防、地方志、民族宗教、社会科学研究、人口与计划生育等工作，都取得新的成绩。
2007年是民生工程步伐明显加快的一年，全年投入财政资金4亿元。积极促进就业再就业，动态消除市区零就业家庭，全年新增城镇就业岗位3.5万个，再就业2万人。继续提高退休金标准，将符合政策规定的城镇未参保集体企业退休人员全部纳入基本生活费补助范围，初步建立失地农民养老保险和农村居民最低生活保障制度，五保供养对象分散和集中供养标准分别达到1200元和1717元。启动五河、固镇和淮上区新型农村合作医疗制度，参合率达89%。建立了城镇居民基本医疗保障制度。对城乡医疗救助对象实施医疗救助。建立了重大传染病病人医疗救治和生活救助保障机制。全部免除学生义务教育阶段学杂费，重视解决进城农民工子女入学问题，免费对农村贫困家庭学生提供教科书并补助寄宿生生活费。改造农村中小学危房5.98万平方米。解决了12万农村群众安全饮水问题。进一步完善农村部分计划生育家庭奖励扶助制度。新增市区公交车辆50辆，线路5条、站点67个。改造城区支路和小街小巷27条，并完成路灯配套。开工建设经济适用房2.2万平方米。改造4个城中村，建设改造21个垃圾中转站、32座城市公厕。广播电视“村村通”覆盖率达到100%。灾区群众基本生活得到保障。
——积极推进改革创新，发展环境继续优化。深化行政审批制度改革，全面加强机关效能建设，改善行政服务中心管理，审批效率继续提高。优化创业环境，兑现优惠政策，全民创业取得新进展，新建立重点创业基地4个，新增私营企业1200家、个体工商户9500户，非公有制经济比重达到45%。主动接受市人大及其常委会监督，积极采纳政协委员和社会各界人士建议，政府重要决策邀请人大、政协领导参与，重大问题向四大班子联席会议通报。认真办理人大代表议案、建议和政协委员提案。加强与民主党派、工商联和人民团体的联系。继续办好政风行风热线、政风行风面对面节目，密切政府与群众的联系。健全突发公共事件应急预案体系。做好信访、法律援助、人民调解等工作，社会矛盾有效化解。推进平安建设，注重安全生产，人民群众安全感增强。推进依法行政，强化监察和审计工作，查处违纪违法案件。完善目标管理和政务督查，促进了各项工作的落实。
2007年各项目标任务的顺利完成，为实现本届政府任期目标画上了圆满句号。过去的五年，在中央和省大政方针指引下，我们积极探索适合蚌埠市情的发展之路，制定并实施了一系列行之有效的战略决策和工作部署，较好应对了复杂的环境变化，战胜了非典、洪水等多种自然灾害和突发事件，取得了新的重大成就。过去的五年，是我市改革开放深入推进的五年，是经济发展和社会进步明显的五年，是人民得到较多实惠的五年，也是城乡面貌发生较大变化的五年。
——五年来，我们坚持以经济建设为中心，经济实力连上新台阶。提出并大力实施工业强市战略，初步形成卧薪尝胆、决战工业之势。统筹城乡发展，扎实有效地推进新农村建设。坚持发展服务业不动摇，巩固区域性商贸中心城市地位。努力培育骨干企业和龙头企业，提高产业竞争能力。狠抓项目和投资工作，全面实施“3461”行动计划，滚动实施283个重点项目。2007年地区生产总值、人均地区生产总值、社会消费品零售总额、进出口总额、固定资产投资、工业增加值、第三产业增加值、财政收入和人均财政收入等主要经济指标，均比2002年翻一番或基本翻一番。地区生产总值连跨300亿元和400亿元台阶，财政收入连续突破30亿元、40亿元和50亿元大关。
——五年来，我们坚持深化改革开放，发展活力明显增强。积极推进国有企业改革，大力发展非公有制经济，初步形成多种所有制经济竞相发展的良好局面。启动农村综合改革试点工作。积极推进公共财政改革、城市管理体制改革和事业单位分类改革。努力创造条件扩大对外开放，与30多个国家和地区建立了经贸联系，与10多个国外城市建立和巩固了友城关系，经济外向度有所提高。招商引资取得积极成果，先后引进中粮集团、中建材集团等一批知名企业，五年引进资金348亿元。
——五年来，我们坚持统筹经济社会发展，社会事业实现新进步。组织实施科技项目463项，转化科技成果136项。义务教育成果更加巩固，高考成绩连年攀升，职业教育同步发展。贯彻教育均衡发展方针，加强薄弱学校建设，推行素质教育。花鼓灯、泗州戏列入首批国家非物质文化遗产保护名录。医疗卫生服务体系进一步完善，食品药品监管得到加强，疾病控制、医疗救治和紧急救援三大公共卫生服务体系基本建立。全民健身活动深入开展，竞技体育水平明显提升。拥军优属、拥政爱民不断创新，荣获全国双拥模范城“四连冠”和全省双拥模范城“五连冠”。
——五年来，我们坚持以人为本，人民生活水平不断提高。努力创造就业岗位，新增就业16.8万人、再就业10.9万人，其中3万名就业困难人员实现了再就业。努力提高社会保障水平，城市低保对象应保尽保，农村低保制度全面铺开。努力提高人民收入水平，城镇居民人均可支配收入、农民人均纯收入年均分别增长11.3%和10.3%，城乡居民储蓄存款人均余额由2002年的3862元增加到6908元。努力改善居住条件，城市人均住房面积由19.1平方米增加到24平方米。
——五年来，我们坚持软件、硬件一起抓，发展环境取得新改善。调整了市区布局，编制完成城市总体发展规划、城市近期建设规划和覆盖城乡的部分专项规划，市区建成区面积增加30平方公里。城市功能逐步完善。实施了一批园林景观建设工程，全市森林覆盖率提高到17.5%。继续加强基础设施建设，城区形成了“七纵七横”主干路网，城外构建了十字交叉的高速公路网，电力、水利等建设取得新成绩。新改建城市道路157公里、省道111公里、县乡道路1839公里。建成合徐、界阜蚌、蚌明高速公路。审批制度改革逐步深化，政府职能有效转变，政策环境更加优惠，被列为比照实施东北地区老工业基地政策范围，怀远县同时享受西部大开发有关政策。
各位代表！过去的五年，我们走过不平凡历程，迈出了新步伐，成绩来之不易。这是中共蚌埠市委正确领导的结果，这是全市人民艰苦努力的结果。在此，我谨代表市人民政府，向全市各族人民，向广大工人、农民、知识分子、干部和解放军指战员、武警官兵、政法干警，向国家和省驻蚌单位，向各民主党派、工商联、各人民团体和其他各界人士，向人大代表、政协委员，向离退休老同志，向在蚌埠创业的境内外投资者和建设者，向所有关心、支持和参与蚌埠现代化建设的海内外朋友，表示崇高敬意和衷心感谢！
在肯定成绩的同时，我们也清醒地看到，发展中仍然存在一些矛盾和问题。主要是：发展速度还不够快，与重振雄风、再创辉煌的要求有差距；发展方式还不够好，经济效益不高，节能减排、保护环境任务繁重；经济结构还不够优，工业主导作用发挥不足，农业产业化水平不高，现代服务业尚在起步，大型企业数量太少；发展后劲和活力还不够强，带动全市发展的重大工业项目不足；城乡居民收入增加还不够多，仍有部分群众生活比较困难，农村公共事业发展相对滞后，就业和社会保障压力比较大；城市建设和管理还不够到位，不文明行为和市区脏乱差现象依然存在；发展环境还不够令人满意，政府依法行政和服务水平有待提高，体制机制性障碍仍然较多，改革的任务还很艰巨等等。对于这些问题，我们要共同努力，认真加以解决。
二、今后五年目标任务
今后五年是全面建设小康社会的关键时期。我们正面临经济全球化深入发展和世界经济总体保持增长的机遇，面临国家促进中部崛起特别是促进老工业基地振兴的机遇，面临沿海地区产业和投资加速向中西部转移的机遇，面临我省激励扶持 “两淮一蚌”城市群发展的机遇，面临全市上下心齐气顺、乘势向上、劲头正足的机遇。我们必须抓住新机遇，迎接新挑战，实现新发展，在新的起点上向全面建设小康社会新目标继续迈进。这是我们肩负的历史使命，也是全市人民的共同愿望。
未来五年政府工作的总体要求是，坚持以科学发展观统领全局，以抢抓机遇、跨越发展、争先崛起为主题，以转变发展方式、促进结构调整为主线，以推进新型工业化、城镇化、农业现代化为重点，以深化改革开放、推进科技创新为动力，以改善民生、构建和谐社会为出发点，深入实施工业强市战略、开放带动战略、科教兴市与人才强市战略、可持续发展战略，继续提升“3461”行动计划，解放思想，扎实工作，努力实现经济社会又好又快发展，夺取全面建设小康社会新胜利，谱写蚌埠人民美好生活新篇章。
今后五年经济社会发展的主要目标和任务是：
（一）加快提升经济实力，努力实现跨越发展。坚定不移地推进工业强市战略，加大决战工业力度，推动工业较快增长。积极发展现代农业和农村经济。改造传统服务业，培育现代服务业。支持骨干龙头企业发展。提高公有制经济水平，大力发展非公有制经济、混合型经济，加快发展县域经济、园区经济。继续加大固定资产投资，五年投入1000亿元以上。通过努力，力争全市地区生产总值、财政总收入五年再翻一番，城镇居民人均可支配收入和农民人均纯收入年均增长7%以上，进一步提升区域性加工、商贸、科技中心城市地位。
（二）加快调整经济结构，切实转变发展方式。坚持走新型工业化道路，坚持以质取胜、质量兴市。按照调优一产、调强二产、调高三产的目标，促进产业集群化发展，全面提升产业层次和水平，推动三次产业快速、联动、集约发展，工业化率达到40%。加大科技创新力度，高新技术产业比重提高到34%，创全国和省名牌产品取得显著进展。降低能源消耗，万元地区生产总值能耗降低20%，COD排放降低9.78%。
（三）继续深化各项改革，提高对外开放水平。推进农村综合改革，完善农业投入政策。搞好国有企业改革重组，继续盘活存量国有资产，化解国有企业改革遗留问题。深化行政管理体制改革，完善政府管理经济的方式和手段，强化社会管理和公共服务职能，提高行政效能，降低行政成本。分类推进事业单位体制改革，力争取得较大突破。鼓励和引导全民创业，支持中小企业发展，增加企业数量、提高企业质量。坚持全方位招商引资不动摇，提高招商引资质量和水平。继续以东向发展为重点，加快接轨和融入长三角经济圈。争取五年累计利用市外资金800亿元以上。 
（四）加强基础设施建设，加快改变城乡面貌。完成村村通公路工程，进一步完善农村电网。基本完成新一轮淮河治理任务，加快中小型水库除险加固，继续完善农村水利设施，提高抗洪排涝能力。完成京沪高速铁路及蚌埠站、合蚌客运专线、水蚌铁路改造等境内铁路建设任务，调整市区铁路布局，建设姜桥物流工业园区。建成蚌埠至淮南高速公路及其通向市区的连接线，建设徐州至明光高速公路蚌埠段和固镇境内高速公路连接线。建成蚌埠闸复线船闸，扩建蚌埠新港，提高通航和港口吞吐能力。建成国电蚌埠电厂一期，争取二期开工，力争建设若干座生物发电厂。建设城市备用水源。完善市区道路交通网络，建成黄山大道、淮上大道、东海大道西延段等骨干道路，建成大庆路淮河大桥，取消市区淮河公路桥收费。实施“东进、西联、南拓、北跨、中优”的空间布局，完善城区功能，拉开城市框架，拓展发展空间。编制和实施村镇建设规划，提升城镇综合承载能力，有序建设农民新居，确保新农村建设取得重要进展。加快城镇规划建设步伐，城镇化率达到40%以上。
（五）加快发展社会事业，促进文明素质提高。坚持教育优先，增加教育投入，均衡发展义务教育，加快普及高中阶段教育，大力发展职业教育，支持发展高等教育，建设全民学习、终身学习型社会。加大科技投入，做好科技人才培养和引进工作，加快建设创新型城市步伐。挖掘历史文化底蕴，保护和抢救物质和非物质文化遗产，打好淮河文化、大禹文化、双墩文化三张牌，舞红花鼓灯，建成市博物馆等重大设施，推动文化事业和产业的发展繁荣。建立覆盖城乡的公共卫生服务体系、药品供应保障体系，完善重大疾病防控体系，加强农村三级卫生服务体系和城市社区卫生服务体系建设。继续搞好人口与计划生育工作，稳定低生育水平，提高人口素质。加强体育工作，建成市龙湖体育馆等设施。开展多种多样的文明市民教育活动，弘扬当代城市精神，争创省级、国家级文明城市。
（六）积极实施民生工程，推进和谐蚌埠建设。坚持就业优先，通过发展经济、培训技能、创业带动、鼓励灵活就业和劳务输出等多种措施，让更多城乡劳动者有就业机会。以社会保险、社会救助、社会福利为基础，以基本养老、基本医疗、最低生活保障制度为重点，以慈善事业、商业保险为补充，加快建立和完善覆盖城乡的社会保障体系。健全覆盖全市的新型农村合作医疗制度和城镇居民医疗保险制度，努力解决群众看病难问题。健全资助制度，保障经济困难家庭、进城务工人员子女等平等接受义务教育。健全廉租住房制度，建设经济适用房，完成市区31个棚户区和旧住宅区改造，加快解决城市低收入家庭住房困难。加大公共交通投入，继续改造小街小巷和公共厕所，增加环卫设施，逐步解决出行难、环境差的问题。我们要继续努力，在经济发展的基础上，真正使全市人民学有所教、劳有所得、病有所医、老有所养、住有所居，推动建设和谐社会。
（七）努力建设生态文明，打造宜居型城市。树立生态文明观，坚持生产发展、生活富裕、生态良好的文明发展道路，建设资源节约型、环境友好型社会。调整优化产业结构和企业布局，禁止建设难以达标排放的水污染项目和其它污染严重的项目，加快老企业退市进园步伐，力争完成八一股份公司老厂区等化工企业搬迁任务，坚决依法关闭长期污染严重又无法改造的企业，大力推进清洁生产，加快发展循环经济，引导企业开展环境认证和其他绿色认证。完善环境监测预警机制，加强生态保护和环境治理，促进经济发展与环境承载能力相适应。加强土地资源调控，加强土地开发、复垦整理，依法保护、合理利用耕地等自然资源。以建设山水园林城市、生态城市为抓手，加快张公山大塘周边环境治理，加快龙子湖、三叉河湿地、沱湖和涂山、锥子山等景区景点建设，加快建成一批城市污水处理厂。大力推进植树造林，提高城市绿地率。提高城市建设和管理水平，努力把蚌埠建设成为适宜人居的淮畔名城。
（八）全面加强社会建设和管理，继续优化发展环境。加强基层组织建设，完善民主管理制度，努力把城乡社区建设成为管理有序、服务完善、文明祥和的社会生活共同体。支持人民团体和群众组织发挥积极作用，切实做好民族、宗教、外事、侨务等工作。推进民主法制建设，开展法制宣传教育，依法规范信访秩序，加快建设法制蚌埠。加强信用体系建设，优化金融生态环境，创造金融安全区，加快建设信用蚌埠。加强信息化建设，构筑覆盖面更广的各类信息网，加快建设数字蚌埠。实施食品药品放心工程，建立健全预防为主的安全生产保障体系，继续完善消防设施，推进社会治安综合治理，依法严厉打击各种违法犯罪活动，保持社会安定有序，加快建设平安蚌埠。
实现上述目标任务，政府责任重大。我们要继续增强为人民服务、为蚌埠崛起作奉献的责任心，发扬勤政、廉政精神和求真务实作风，加快行政管理体制改革，建设服务型政府，努力开创政府自身建设新局面。只要全市上下万众一心，团结奋斗，五年后的蚌埠就一定会更加繁荣美好。
三、2008年工作安排
千里之行，始于足下。2008年是新一届政府开局之年，是实施“十一五”规划的关键之年，必须开好头起好步。今年经济社会发展的主要预期目标是：地区生产总值增长12%以上，一、二、三产分别增长5%、16%和12%，全部工业增加值增长16.5%以上;财政总收入增长12.3%，其中地方财政收入增长13.5%；固定资产投资增长20%；利用境外资金增长15%，引进市外资金增长30%；社会消费品零售总额增长14%；进出口总额增长11%；城镇居民人均可支配收入和农民人均纯收入均增长7%；城镇登记失业率、人口自然增长率、居民消费价格涨幅控制在省下达的目标以内；万元地区生产总值能耗降低4.4%。
为了完成上述目标，必须切实抓好以下十个方面的工作：
（一）促进工业加快发展
加快工业发展十分关键，必须继续落实优先发展工业的各项政策，加大工作力度，确保实现预期目标。一是继续培育大企业。支持和引导产品有市场、发展有潜力、竞争有优势的企业内引外联，做大做强。加快形成骨干企业群，力争新增规模以上工业企业80户，年销售收入过亿元企业达到40户。积极培育和引导专精特新中小企业加快发展。二是继续实施大项目。重点抓好150个千万元以上工业项目，积极推进新开工项目前期工作，加快续建项目实施进度，力争建成50个千万元以上重点工业项目，全年完成工业投资92亿元。加快建设昊方公司1000万套汽车空调电磁离合器、国瑞特公司重型机床、丰原集团3000吨Vc、丰原药业6000万瓶塑料瓶输液、雷诺公司二氧化锡导电膜玻璃、华益公司50万片抗反射膜玻璃等项目。尽快建成昊业公司3000万只高效滤清器、液力机械厂油缸变矩器扩产改造、中艺公司年产3万吨钢结构及彩色压型钢板、八一化工5000吨愈创木酚、贝迪斯公司高精密金属膜电阻器、拓普森公司40万只高倍率锂电池、华润公司10万吨啤酒、创业公司5万锭纺纱等项目。开工建设宏业集团7万锭纺纱设备技改、鑫润公司高档织物、新奥公司10万吨二甲醚、科达公司3万台光电仪器及检测设备等项目。 三是继续加快工业园区建设。全面实施6个省级开发区近期规划，加快编制远期规划，拓展园区发展空间。支持产业基础较好、功能布局合理的省级开发区规划创建循环经济园区。推进高新区申报国家级开发区工作。加快市区3个工业集中区和重点乡镇工业集中区建设。进一步推进凯盛玻璃新材料产业园、滤清器产业园、精细化工园等各类特色专业园区的建设和发展。加快建设省环保设备产业基地。研究扶持产业集群发展的政策措施，促进滤清器、电子元器件、玉器加工、服装加工等具有一定发展基础的特色优势产业加快集聚。四是继续提升自主创新能力。引导企业加大科技投入，积极建设国家级和省级技术中心，组织开展多种形式的产学研协作活动，提高企业新产品研发能力，完成新产品开发180项。实施品牌带动战略，争创名牌产品、著名商标5个，增强市场竞争力。五是继续提高为工业服务水平。强化工业经济运行监测和分析，加强煤电油运的调度，搞好协调服务，保障工业经济正常有序运行。加强对县区和重点企业考核，调动各方面发展工业的积极性。
（二）切实做好“三农”工作
做好“三农”工作是重中之重。要以农业增效、农民增收为核心，加快发展现代农业，促进农村经济发展，推进新农村建设。一是大力调整农业结构，提高农业质量和效益。鼓励发展特色农业，因地制宜建立产业集中区。继续实施小麦高产攻关和水稻产业提升行动，争取实施玉米振兴计划，推广种植粳糯稻90万亩。扩大无公害、绿色、有机农产品生产规模，新增无公害农产品基地10万亩。提高养殖业规模和比重，新建养殖小区20个。二是大力推进农业产业化，提高组织化程度和技术水平。以发展工业理念推动农产品深加工，促进农产品加工升值。做大做强95 家市级以上龙头企业，整合规范专业合作经济组织。加快农业先进适用技术、优良品种的引进和推广，农机化综合水平提高2个百分点以上。大力推广标准化，认证安全农产品10个。三是大力加强农业综合开发，继续增加农业投入。争取实施更多的农业开发项目。完成芡河、跃进沟、黑泥沟洼地治理，实施樵子涧中型水库和14座小水库除险加固。推进农田水利工程，改造中低产田14万亩。完善林权制度改革。完成成片造林1万亩，新建绿色长廊30公里。四是落实农村政策，拓宽农民增收渠道。认真落实良种补贴、农机补贴等惠农政策。继续实施农村劳动力转移培训“阳光工程”，帮助农民转移就业。积极培育物流、信息、咨询、中介等服务市场，引导农民就业创业。推进畜牧体制改革，加快动物疫病防控体系建设。开展放心农资下乡活动，保护农民利益。五是支持县域经济发展，壮大县域经济实力。继续实施工业强县战略，促进农村工业、农村流通业和现代服务业加快发展，构筑有特色的产业集群。依托重点城镇和工业园区，改善投资环境，强化协调服务，大力推进招商引资。完善县域经济考核机制，建立奖惩激励制度，推动县域经济更好更快发展。
（三）着力发展第三产业
服务业潜力很大，必须加快发展。要继续改造和提升传统服务业，同时积极培育新兴服务业；继续改造传统商业区，同时培育新兴商业区。巩固老城区商业中心地位，编制完成淮河路商业街区规划，启动淮河路步行街改造，开发二马路商业片区，激活市区“两站”商业圈。开工建设合百商厦、沃尔玛购物中心、商之都、苏果超市等重点项目。积极谋划高铁站周边商业圈。加快新城中心商务区建设，宝龙广场主体竣工，华夏商业步行街投入运营，打造淮河文化广场商务服务中心。加快建设淮上商贸新区，支持通成国贸广场、路桥市场扩大经营。拓展农村市场，推进“万村千乡”市场工程和“新网”工程，加大农家店的建设和改造力度。整顿和规范市场经济秩序，加强市场监管，依法打击传销。加快发展现代物流业，争取开工建设蚌埠新港二期等项目，加速推进蚌埠闸船闸扩建、姜桥物流工业园区、光彩大市场二期、现代物流园区等项目建设。继续促进房地产业稳步发展。积极培育会展、金融保险、证券期货和信息咨询等服务业。力争在服务外包等方面取得进展。加快发展旅游产业。启动梅花山、大巩山景区建设项目，推进涂山、锥子山、东—西芦山和垓下古战场等景区景点建设。争创2个4A级景区、1个国家农业旅游示范点。建成并开放孙家圩子渡江战役总前委旧址，发展红色旅游，开发新的旅游线路。促进旅游纪念品生产，加快建设蚌埠工艺美术产业园。
（四）继续扩大项目投资和招商引资
坚持实施投资带动战略不动摇，扩大投资规模，改善投资结构，提高投资质量。深入实施“3461”行动计划，完善重大项目领导分工联系制度，加强项目调度和督查，强化目标分解考核，不断充实“3461”项目库，确保重点项目完成投资占全部投资的40%以上。加强重点项目跟踪服务，力争年内建成丰原集团玉米秸秆和玉米芯综合加工、八一3000吨香兰素等项目；继续加快国电蚌埠电厂、汽配产业园南园、丰原生物质产业、普乐新能源等项目建设，确保按期完成序时进度；积极落实各项条件，全面开工建设城市环境综合整治世行项目、水环境综合治理日元项目、大庆路淮河大桥及五河、固镇秸秆发电厂等一批重点项目；积极推进淮干河道整治、行蓄洪区调整等重大项目前期工作；扎实做好京沪高速铁路蚌埠段、徐明高速公路蚌埠段、蚌淮高速公路等国家、省重大项目的衔接和建设保障工作。建立健全项目谋划工作目标责任制、激励机制和推进机制，重点推进汽车工业园、船舶工业园、电子产业园等在手谋划项目，超前谋划、储备一批有牵动性的重大加工制造业项目，力争新增项目储备规模200亿元。加大资金争取力度，落实国债及各类专项资金。继续举办银企对接活动，争取商业银行的更多支持。继续加强与国家开发银行的新一轮合作，尽快落实申贷额度。推进企业上市，支持上市公司再融资。争取发行企业债券。加强与保险资金和各类投资基金合作，开创融资工作新局面。
促进招商引资不断升温，力争在资金数量和项目质量方面取得新突破。继续强化领导责任，加强招商引资目标考核。继续改进驻点招商，全面推进专职招商，充实招商力量，提高招商实效。积极组织参加全国性及省里统一安排的招商活动，重点组织好与长三角、珠三角地区合作恳谈会等主题招商活动。落实重点招商项目，分类跟踪，全程服务，确保在谈项目谈成，谈成项目落地，督促资金按时到位，招商引资成果落到实处。坚持“挑商选资”原则，防止引入有损生态环境的项目。
（五）推进改革开放和全民创业
继续加快各项改革步伐。深化国有资产管理体制改革，建立国有资本经营预算制度。运用市场化手段推动国有存量资产向国有资本转变，实现收益最大化。完成市属国有企业破产案件收尾工作。深化收入分配制度改革，建立企业职工工资正常增长和支付保障机制。深化财政体制改革，增强公共财政透明度。全面推进农村综合改革，完善农村“一事一议”公益事业财政奖补政策，推动建立透明、高效的农村基层管理体制和运行机制。积极稳妥地推进科技、教育、卫生、医疗、文化等社会事业管理体制改革。
继续扩大对外开放。加快实施东向发展战略，发挥比较优势，主动对接长三角经济圈，在引资引智、产业融合、要素配置等方面实现新突破。积极参与“两淮一蚌”城市群和淮海经济区建设，推动区域经济协调发展。进一步转变外贸增长方式，坚持以质取胜，大力推进丰原柠檬酸、金威滤清器、八一顺达对（邻）硝基氯化苯等自主品牌产品出口。深入实施科技兴贸战略，落实出口促进政策，加快国家级科技兴贸出口创新基地和汽车零部件出口基地企业建设，提高高新技术和机电产品出口份额，扩大加工贸易比例。加强对外合作与交流，巩固和发展友城关系。
继续鼓励全民创业，促进非公有制经济发展。清理和修订各种不利于非公有制经济发展的规定。实行公平的市场准入原则，引导和鼓励非公有制企业参与建设公用事业、基础设施、社会事业等方面的项目。完善融资担保体系，改进对非公有制经济的服务方式。进一步营造全民创业氛围，全面落实鼓励全民创业的政策措施，积极建设创业服务体系和创业平台，努力形成不拘形式谋创业，敢为人先创事业的生动局面。今年要力争再新增个体工商户5800户、私营企业1280家，非公有制经济占国民经济的比重要增加到47%以上。
（六）继续强化城市建设和管理
坚持城市建设规划先行方针，完善城市规划体系，做到按规划建设。完善城市中心地区、旧城改造地区、近期发展地区等控制性详细规划，引导城市开发建设活动。组织开展淮河风光带及两岸滨河地区规划设计。编制和完善城市绿地控制系统、环卫设施定点布局、人防工程布局等专项规划，组织编制城市生态内环景观、市域道路系统规划和城市分区规划。完成解放路南段、燕山路东段改造，实施南山路中段、凤阳路西段拓宽改造，打通体育路东段，延伸延安路南段，启动延安路穿铁路箱涵扩孔工程。建设龙子湖西岸地区路网，加快淮上新区道路建设和光彩客运南站建设。继续建设黄山大道。加快城市圈堤改造，开工建设八里沟西堤防洪工程，建成圈堤新船塘段。实施城市供水管网和供水水质改造项目，铺设天然气管道20公里。继续实施“四路一桥”环境和城市出入口综合整治工程。加快张公山大塘、龙子湖周边地区整治和综合开发，建成城市圈堤公园等，启动花卉苗木基地建设。加快旧城改造步伐，推进31个棚户区、旧住宅区改造，基本建成大板楼危房改造安置房工程。加大经济适用房建设力度，完善廉租住房制度。
坚持城市管理规章先行方针，按规章加强城市管理。健全科学明确的城市管理责任体系，构建协调有序的部门联动工作机制，推进管理重心下移和事权下放，提高城市管理综合执法效能。狠抓市容环境综合整治，开展专项整治活动，树立文明的城市形象。加强城区环境卫生管理，建立新的环卫体制和运作机制。抓好违法建筑的防控和拆除工作。加强建设工程管理，整顿规范建筑市场秩序。城市管理要在目前基础上，再上一个新台阶。县城管理也要有明显加强和进步。
（七）加强资源节约和环境保护
切实增强环境承载能力。进一步健全环境监管体制，提高环境监管、应急预警和重大环境污染事件处置能力。加大环保执法力度，完成海兴化工等5家化工企业搬迁改造任务。抓好重点污染企业污染控制，确保重点工业污染源达标排放率在95%以上，COD削减2.44%以上。建成第二、第三城市污水处理厂并投入使用，启动城南污水处理厂项目，完善城区污水管网配套，城市污水集中处理率达50%以上。综合防治农业面源污染和畜禽养殖污染。落实淮河、天河等饮用水源地保护措施，推进城市东部供水工程，完善应急处置机制，确保饮用水安全。加快建设医疗卫生废物集中处置基地。开展空气污染环境综合治理，大力推进燃煤脱硫工程，全年好于Ⅱ级空气天数要达到95%以上。加强各类噪声源的监控与管理，扩大城镇噪声达标区范围。从严查处环境污染扰民问题，维护人民群众的环境权益。切实加强资源节约利用。推广应用节能环保新技术、新工艺、新设备，加速淘汰落后生产能力。落实促进循环经济发展的各项政策措施，加快发展和利用可再生、无污染的清洁能源。突出抓好重点领域、重点产品、重点企业节能降耗工作，力争规模以上工业企业万元增加值综合能耗降低4.4%以上。推广高性能、低耗材、可循环的建筑材料，提高新型墙体材料应用比重。坚持节约集约用地，严格控制和合理开发利用土地资源，提高投资强度和产出效益。加大闲置土地清理力度，盘活存量土地。加强耕地保护，加大土地开发整理力度，整理新增耕地400公顷。
（八）促进各项社会事业协调发展
加大科技创新力度，完善科技创新平台建设。筹建大学科技园区。制定市区中小学布局调整规划，完成义务教育阶段学校招生区域重新划分工作。落实义务教育经费保障机制。发展非义务阶段教育，适度扩大高中办学规模，整合职业教育资源，支持发展高等教育。促进民办教育事业健康发展。规范医疗服务市场，加强疾病预防控制和公共卫生服务，提高应急救治能力。加强人口与计划生育工作，统筹解决人口问题。深入开展全民健身运动，保持竞技体育实力。积极发展文化事业和文化产业。加快农村文化设施建设步伐，不断巩固和壮大城乡基本文化阵地。创作艺术精品，丰富城乡群众文化生活。做好双墩遗址和禹墟的发掘工作，举办双墩出土文物展。推进档案馆、新博物馆建设准备工作，启动二实小东校区建设，支持二中新校区、三院新院区等重点工程建设，完成广播电视信息数据平台建设。认真落实促进就业和再就业的政策措施，努力拓宽就业渠道。不断完善社会保障体系，强力推进社会保险参保扩面工作，加强社会保险基金的征收管理。高度关注弱势群体生活，完善对残疾人家庭、城市困难居民和农村五保户、特困户的经常性救助体系。启动市流浪未成年人救助保护中心建设。加强群众来信来访工作，积极预防和妥善处理群体性事件。加强国防教育和建设，扎实做好“双拥”工作。强化社会治安综合治理，完善防控体系建设。严格落实安全生产责任制，坚决遏制重特大事故的发生。
（九）大力实施民生工程
民生问题始终是推进和谐社会建设的重中之重。要贯彻尽力而为、量力而行原则，每年都在解决民生问题上做更多实事。今年的重点是：1、继续解决就业难问题，新增城镇就业岗位3.2万个。2、继续做好社会保障工作，扩大农村低保覆盖面，提高城乡居民最低生活保障标准，统筹管理未参保集体企业退休人员生活保障金并实行按月发放。3、继续解决看病难、看病贵问题。努力实现城镇居民医疗保险全覆盖；确保新型农村合作医疗全覆盖，农民参合率达到90%；新建改建111所农村卫生室和27个社区卫生服务中心（站）；提高城乡医疗救助标准，实施重大传染病医疗和生活救助；建立城市困难群众医疗费减免和政府补贴制度；逐步扩大社区卫生免费服务范围。4、继续支持发展义务教育，改造4万平方米农村中小学危房。5、帮助解决城市困难群众住房问题，建设陶店等处的20万平方米经济适用房和2万平方米廉租房。6、继续改善农民生产生活条件，启动13个新农村示范镇村建设，新改建农村公路500公里、农村公路客运站8个，实施渡改桥工程5个，解决12万农民安全饮水，完成新一轮淮河行蓄洪区移民迁建3400户。7、继续解决城市居民出行难、居住环境差等问题，安装更换市区50条道路及小街小巷路灯；推行公交IC卡消费管理系统，新增公交线路2条、站点40个；建设改造2座垃圾转运站和25座市内公厕。8、继续关爱老年人，新建改建10所农村敬老院，集中供养率提高到25%。9、支持计划生育，继续对2000个农村部分计划生育家庭实施奖励扶助。10、支持体育健身活动，为36个行政村配备健身器材。随着经济发展，我们要把财政资金更多地投入到社会发展薄弱环节上来，投入到解决群众切身利益问题上来，让经济发展成果更多地体现到改善民生上来。
（十）努力提高政府工作水平
进一步增强服务发展能力。继续转变政府职能，加强机关效能建设，深化行政审批制度改革，强化行政服务中心功能，提高服务水平。加大招投标统一平台和电子政务建设，健全网上审批系统，提高办事效率。创造性地贯彻国家宏观政策，做好土地、金融、环保、财税等工作，积极化解经济发展中的矛盾。更加注重民生问题，更加注重生态建设，更加注重加强社会管理、改善公共服务。进一步提高依法行政能力。规范政府行为，积极稳妥地推行综合行政执法试点工作，探索相对集中行政许可权试点，严格依法行政。坚持政务公开，重大决策和事项广泛征求意见，确保行政权力透明运行。依法向人大及其常委会报告工作、向政协通报情况，自觉接受人民群众的社会监督、新闻媒体的舆论监督，支持政协和各民主党派、工商联、人民团体参政议政。进一步增强政府执行能力。理顺市县两级、政府各部门之间的层级关系，合理界定职能边界，建立部门间的联席会议制度，形成执行顺畅、合力攻坚的机制。完善政府责任分解机制，创新完善科学的考评机制，严格执行行政监督、责任追究、纠错改正制度，增强执行政策的坚定性、一贯性、效率性。进一步提高廉洁自律能力。加强作风建设，强化勤政、廉政的自觉性。进一步健全惩防腐败体系，着重从源头上预防和治理腐败，全面落实廉政建设责任制。强化审计监督、行政监察和社会监督，严肃查处违纪违法行为。加强节约型机关建设，厉行勤俭节约，力戒铺张浪费，严格控制机关楼堂馆所建设，规范公务接待活动，切实降低行政成本。
各位代表！实现科学发展、和谐发展、跨越发展，加快全面建设小康社会进程，是我们共同的目标和责任。让我们在中共蚌埠市委的坚强领导下，高举中国特色社会主义伟大旗帜，弘扬“禹风厚德、孕沙成珠、务实开放、创业争先”的城市精神，凝心聚力，团结拼搏，为圆满完成今年和今后五年目标任务，为实现蚌埠早日崛起和人民安康富裕而继续努力奋斗！
